# Association analysis of *PRNP *gene region with chronic wasting disease in Rocky Mountain elk

**DOI:** 10.1186/1756-0500-3-314

**Published:** 2010-11-18

**Authors:** Stephen N White, Terry R Spraker, James O Reynolds, Katherine I O'Rourke

**Affiliations:** 1 Animal Disease Research Unit, Agricultural Research Service, U.S. Department of Agriculture, Pullman, WA 99164, USA; 2 Department of Veterinary Microbiology and Pathology, Washington State University, Pullman, WA 99164, USA; 3 Center for Integrated Biotechnology, Washington State University, Pullman, WA 99164, USA; 4 Colorado State University College of Veterinary Medicine & Biomedical Sciences, Fort Collins, CO 80526, USA

## Abstract

**Background:**

Chronic wasting disease (CWD) is a transmissible spongiform encephalopathy (TSE) of cervids including white-tailed (*Odocoileus virginianus*) and mule deer (*Odocoileus hemionus*), Rocky Mountain elk (*Cervus elaphus nelsoni*), and moose (*Alces alces*). A leucine variant at position 132 (132L) in prion protein of Rocky Mountain elk confers a long incubation time with CWD, but not complete resistance. However, variants in regulatory regions outside the open reading frame of *PRNP *have been associated with varying degrees of susceptibility to prion disease in other species, and some variants have been observed in similar regions of Rocky Mountain elk *PRNP*. Thus, additional genetic variants might provide increased protection, either alone or in combination with 132L.

**Findings:**

This study provided genomic sequence of all exons for *PRNP *of Rocky Mountain elk. Many functional sites in and around the *PRNP *gene region were sequenced, and this report approximately doubled (to 75) the number of known variants in this region. A haplotype-tagging approach was used to reduce the number of genetic variants required to survey this variation in the *PRNP *gene region of 559 Rocky Mountain elk. Eight haplotypes were observed with frequencies over 1.0%, and one haplotype was present at 71.2% frequency, reflecting limited genetic diversity in the *PRNP *gene region.

**Conclusions:**

The presence of 132L cut odds of CWD by more than half (Odds Ratio = 0.43; P = 0.0031), which was similar to a previous report. However after accounting for 132L, no association with CWD was found for any additional variants in the *PRNP *region (P > 0.05).

## Background

Chronic wasting disease (CWD) is a transmissible spongiform encephalopathy (TSE) of cervids including white-tailed and mule deer (*Odocoileus virginianus *and *O. hemionus*, respectively), Rocky Mountain elk (*Cervus elaphus nelsoni*), and moose (*Alces alces*) [1]. This invariably fatal disease can be associated with progressive physical wasting, often accompanied by behavioral changes including listlessness, decreased interaction with other animals, decreased responsiveness to environmental stimuli, and repetitive behaviors [1]. There is no cure for CWD or any of the TSEs, and current management practice to reduce the spread of CWD includes depopulation or permanent quarantine of infected farmed herds as well as removal of free-ranging animals showing clinical signs of disease. Despite these efforts, prevalence continues to climb and the range of CWD continues to expand [2,3].

The TSEs are believed to be caused by a misfolded prion protein (PrP^d^) that is protease resistant and infectious [4]. The prion protein is highly conserved and the normal cellular form of the protein (PrP^c^) is expressed in neurons of all mammals studied to date [5]. The disease form PrP^d ^is capable of recruiting the normal cellular form of the protein (PrP^c^) into the same misfolded conformation [4]. While there is no cure for CWD, genetic variants in and around the prion gene confer varying degrees of resistance to TSEs in a range of species. In sheep, an arginine substitution at position 171 (171R) confers strong resistance to classical scrapie, the TSE of sheep and goats [6]. Scrapie eradication programs in the U.S. and Europe include a large component of breeding animals toward resistant genotypes, and classical scrapie prevalence is in decline [7,8].

Genetic solutions may be possible for Rocky Mountain elk, and a leucine variant at position 132 (132L) in prion protein confers a long incubation time with CWD, but not complete resistance [9-11]. However, additional genetic variants might provide increased protection, either alone or in combination with 132L. Genetic variants in regulatory regions outside the open reading frame of the prion gene have been associated with susceptibility to or incubation time with prion disease in cattle [12], and some genetic variants have been observed in similar regions of Rocky Mountain elk [9,13,14].

The many variants in any gene region are arranged in only a limited number of haplotypes, or linear arrangements of genetic variants as they exist on chromosome segments in a population. To find an important genetic variant, one need only genotype a limited set of variants that "tag" all the major haplotypes in a gene region to capture all or most of the information contained in the complete set of genetic variants in a region [15]. This strategy is known as haplotype-tagging, and it often reduces the number of variants that need to be genotyped by 50-75% or more, greatly reducing the time, cost, and complexity of analysis required to test a region for important genetic variants [16,17]. Importantly, because any disease-related variant will occur on one or more haplotypes in the population, haplotype tagging allows the testing of haplotypes for association with disease whether or not the underlying causative variants are in the dataset, or even known at all. This has given rise to a two-stage strategy for assessing genetic association: first, a relatively small number of individuals are genotyped to determine common haplotypes in a population, and then all individuals are genotyped with only a set of haplotype-tagging markers identified in the first round [16]. Therefore, this study used a two-stage haplotype-tagging strategy to search for any variants in the *PRNP *gene region - whether previously known, discovered in this study, or as yet undiscovered - that may provide increased protection from CWD in Rocky Mountain elk.

## Methods

A total of 559 captive and free-ranging Rocky Mountain elk were sampled from herds following positive CWD diagnosis in 6 states including Colorado, Montana, Minnesota, Nebraska, Oklahoma, and South Dakota. All animal procedures used were exempt by the Institutional Animal Care and Use Committee of Washington State University as no live animal use was involved; all samples were from depopulation programs approved by federal and state regulatory bodies to control the spread of CWD. In total, 120 animals tested positive and 439 tested negative for CWD by immunohistochemical methods as previously described [10]. Briefly, formalin fixed tissues including brain, retropharyngeal lymph node, and tonsil were processed for PrP^d ^detection using an automated monoclonal antibody immunohistochemistry assay [10]. The standard for considering an animal CWD positive was at least one tissue positive for PrP^d^. Because the time between infection and appearance of detectable PrP^d ^is unknown [3], elk from herds exposed to CWD but individually lacking detectable PrP^d ^were not defined as CWD negative; these animals were defined as herd-matched controls.

### Sequence and Association Analysis

Genetic variant discovery was performed on a group of 20 animals chosen for geographic diversity, including animals from Colorado, Montana, Minnesota, and Oklahoma. PCR was performed using primers as shown (additional file 1: amplification primers and conditions) and the following standard PCR conditions: an initial denaturation at 95 degrees for 5 minutes, followed by 35 cycles of 95 degrees for 30 seconds, the annealing temperature (see additional file 1) for 30 seconds, and 68 degrees for 90 seconds, and a final extension step at 68 degrees for 10 minutes. Sequencing employed BigDye chemistry (Applied Biosystems, Foster City, CA) and primers as shown (additional file 2: sequencing primers). The regions sequenced included the promoter, 5' UTR, entire open reading frame, 3' UTR, all exon splice sites, and flanking regions located approximately 20 kb upstream of the sequenced promoter region and approximately 20 kb downstream of exon 3. Deeper resequencing using a subset of these primers was performed on a group of 93 animals (46 cases, 47 controls) to identify additional variants including lower frequency alleles that may be associated with CWD.

Haplotype tagging markers (Table 1) were chosen using an r^2 ^threshold of 0.8 in Tagger as implemented in HAPLOVIEW [18] to represent every variant present at greater than 5% minor allele frequency in either the geographic diversity group of 20 or in the case-control group. Genotyping assays were designed for these haplotype-tagging markers (Tables 2, 3), and genotyping was performed on the full animal set as previously described [19]. Briefly, fluorescent TaqMan genotyping assays were performed according to manufacturer specifications, including standard cycling conditions, (Applied Biosystems, Foster City, CA) using primer and probe sets as shown in Table 2. Restriction fragment length polymorphism (RFLP) assays were performed using standard PCR conditions (see above) and restriction enzymes listed in Table 3 according to manufacturer specifications (New England Biolabs, Ipswich, MA; Fermentas Inc., Glen Burnie, MD; Roche Diagnostics, Indianapolis, IN), and visualized on 2% (mass/volume) agarose gels. One haplotype-tagging marker (g.152T > C; [dbSNP:ss115456962]) was not suitable for either fluorescent or RFLP genotyping assays, so allele-specific PCR was performed to identify genotypes for this marker. The reaction to detect the C allele of this marker used standard PCR conditions (above) with a 51 degree annealing temperature, and employed the following primers: CTAGGTGGAATCAGTCGYAC and GGACTTTGCCCAGAGGGTAG. The reaction to detect the T allele was similar except for a 61 degree annealing temperature, and it employed primers CTAGGTGGAATCAGTCTYAT and GGACTTTGCCCAGAGGGTAG. As with RFLP assays, the results were visualized on 2% (mass/volume) agarose gels. The threshold for successful marker genotyping was genotypes on at least 95% of animals. Haplotype frequencies were calculated using PHASE v2 (Table 4) [20,21]. The threshold for successful genotypic markers was genotype identification in at least 95% of animals with at least 95% confidence in PHASE v2.

**Table 1:** Haplotype-tagging markers genotyped in *PRNP *gene region.

*Marker*	*dbSNP accession*	*Sequence variant*	*Minor Allele*	*Minor Allele Frequency (%)*	*Impact on PRNP*
1	ss115456950	g.371T > C^1^	C	15.1	
2	ss115456954	g.986A > G^1^	G	15.3	
3	ss119994796	g.598T > C^2^	T	23.4	
4	ss119994799	g.655C > T^2^	T	1.3	
5	ss119994806	g.918A > G^2^	G	14.8	
6	ss119994811	g.1131C > G^2^	G	13.4	
7	ss119994818	g.1456C > G^2^	G	8.0	
8	ss119994819	g.1477C > T^2^	T	23.5	
9	ss119994821	g.1591T > C^2^	C	13.9	
10	ss119994830	g.17361G > A^2^	A	23.9	
11	ss119994833	g.17918T > G^2^	T	24.3	
12	ss119994840	g.18059C > T^2^	T	22.4	Silent^4^
13	ss119994835	g.18308G > A^2^	G	8.5	Silent^4^
14	ss119994836	g.18390A > T^2^	T	13.4	L132M^4^
15	ss115456958	g.3C > T^3^	C	13.3	
16	ss115456962	g.152T > C^3^	T	21.9	
17	ss115456969	g.1104C > T^3^	C	8.3	
18	ss119994818, ss119994835	g.1456C > G^2^, g.18308G > A^2^	CG	8.5	
19	ss119994818, ss119994819	g.1456C > G^2^, g.1477C > T^2^	CT	23.3	
20	ss115456954, ss119994833, ss119994836	g.986A > G^1^, g.17918T > G^2^, g.18390A > T^2^	GTT	11.6	
21	ss119994796, ss119994806	g.598T > C^2^, g.918A > G^2^	CA	23.6	
22	ss119994796, ss119994806	g.598T > C^2^, g.918A > G^2^	TG	14.3	

^1^Nucleotide positions in reference to GenBank:FJ590753.

^2^Nucleotide positions in reference to GenBank:FJ590751.

^3^Nucleotide positions in reference to GenBank:FJ590752.

^4^Deduced amino acid substitution.

**Table 2:** Fluorescent Genotyping Assays.

*Sequence variant*	*Reagent Role*	*Oligonucleotide*
g.655C > T^2^	Primer	GCCTGGACTCCACCTTAGG
	Primer	CGATGAGTAACCTGGATACGATCAA
	Probe^4^	TTCTGTTGTCGTTTCCT
	Probe^5^	TCTGTTGTCATTTCCT
g.1131C > G^2^	Primer	GCTACAACAGACTGACTGGATAGAA
	Primer	GGAATGAAGATGATTCAGTAATGGAAATGG
	Probe^4^	CCAGTTAGAAATCAGTATTA
	Probe^5^	CCAGTTAGAAATGAGTATTA
g.18390A > T^2^	Primer	GCAGCTGGAGCAGTGGTA
	Primer	CAAAATGTATAAGAGGCCTGCTCATG
	Probe^4^	TTCCCAGCATGTAGCC
	Probe^5^	CCCAGCAAGTAGCC
g.1456C > G^2^	Primer	GGAGCCTCTTGCCCATAACAA
	Primer	AGAAAACAGGTGTTAGAGGGATCATATACA
	Probe^4^	ATTTATGCTGGGTCAATG
	Probe^5^	TTATGCTGCGTCAATG
g.17361G > A^2^	Primer	TCTGTGCCAGGCTCTGC
	Primer	AGGGAATGAGTCTCTTTTGTTTGCT
	Probe^4^	CCTGGCCCACTAAGA
	Probe^5^	CTGGCCCGCTAAGA
g.1104C > T^3^	Primer	CCATGTGGAGGCCAGAAATCC
	Primer	GATGCCAGAAGAGTTTACAGAGACA
	Probe^4^	CTGGAGGTGGGAAAC
	Probe^5^	CTGGAGGTAGGAAAC
g.986A > G^1^	Primer	ATCCCCTACCTATGACTTTCAGACA
	Primer	TCCCCATAACCCGGAATCCT
	Probe^4^	ATCATAGGCTACTACCTCATT
	Probe^5^	TAGGCTACTGCCTCATT
g.371T > C^1^	Primer	GGACTGTCTAGATGAGCCAAACAT
	Primer	AGAGTCCCAGTTGCAGCTTAG
	Probe^4^	AGGATGACCGAAATAT
	Probe^5^	AGGATGACCAAAATAT
g.18308G > A^2^	Primer	CACATGGTGGTGGAGGCT
	Primer	GCTCCTGCCACATGCTTCA
	Probe^4^	TCAGTGGAACAAACCCAGT
	Probe^5^	AGTGGAACAAGCCCAGT

^1^Nucleotide positions are based on GenBank:FJ590753.

^2^Nucleotide positions are based on GenBank:FJ590751.

^3^Nucleotide positions are based on GenBank:FJ590752.

^4^Taqman probe labelled with VIC dye. ^5^Taqman probe labelled with FAM dye.

**Table 3:** Restriction Fragment Length Polymorphism Assays

*Sequence variant*	*Reagent Role*	*Reagent*	*Annealing Temp*
g.598T > C^2^	Primer	CGAACATGCTTGTCATTCAGT	58
	Primer	ATTTATGGCCTGTCTGGAGGA	
	Enzyme	Bcl I	
g.17918T > G^2^	Primer	GCAAAATCTTGCCTGTTTCC	58
	Primer	GGGGAGGAGAAGAGGATCAC	
	Enzyme	Nsi I	
g.18059C > T^2^	Primer	GCAAAATCTTGCCTGTTTCC	58
	Primer	GGGGAGGAGAAGAGGATCAC	
	Enzyme	Bsa HI	
g.3C > T^3^	Primer	CCACAGAGCCACAGAGCTGCC	63.7
	Primer	GGGCTGGGTGAATTGTGTCATCCT	
	Enzyme	Hinc II	
g.1477C > T^2^	Primer	GGAGCCTCTTGCCCATAACA	63
	Primer	CTACTACCCTGGATTGAGCG	
	Enzyme	Rsa I	
g.1591T > C^2^	Primer	CAAATTTGGGTAATGATGTA	56
	Primer	TTCTACTACCCTGGATTGAG	
	Enzyme	Mae III	
g.918A > G^2^	Primer	CCTGTCGGAGTGTAACCTTGGGC	63
	Primer	TGAGGGAGCTGAGAGGCCAGG	
	Enzyme	Bcc I	

^1^Nucleotide positions are based on GenBank:FJ590753.

^2^Nucleotide positions are based on GenBank:FJ590751.

^3^Nucleotide positions are based on GenBank:FJ590752.

**Table 4:** Haplotypes with frequencies > = 1.0% as determined by PHASE.

*Haplotype*	*1*	*2*	*3*	*4*	*5*	*6*	*7*	*8*
Marker								
g.371T > C^1^	T	C	T	C	T	T	T	C
g.986A > G^1^	A	G	A	G	A	A	A	G
g.598T > C^2^	C	T	T	T	C	C	T	T
g.655C > T^2^	C	C	C	C	T	C	C	C
g.918A > G^2^	A	G	A	A	A	A	G	G
g.1131C > G^2^	C	G	C	C	C	G	G	C
g.1456C > G^2^	C	C	G	G	C	C	C	C
g.1477C > T^2^	T	C	C	C	T	T	C	C
g.1591T > C^2^	C	T	C	C	C	C	T	T
g.17361G > A^2^	A	G	G	G	A	A	G	G
g.17918T > G^2^	G	T	T	T	G	G	T	T
g.18059C > T^2^	C	T	T	T	C	C	T	C
g.18308G > A^2^	G	G	A	A	G	G	G	G
g.18390A > T^2^	A	T	A	A	A	A	T	A
g.3C > T^3^	C	T	C	C	C	C	T	C
g.152T > C^3^	T	C	C	C	T	T	C	T
g.1104C > T^3^	C	C	T	T	C	C	C	C

Haplotype Frequency (%)	71.2	10.1	6.0	1.4	1.2	1.0	1.0	1.0

Haplotype Frequency in captive animals (%)	70.6	10.3	6.2	1.1	1.2	1.1	1.1	1.1

Haplotype Frequency in free-ranging animals (%)	78.6	7.4	4.3	4.2	0.0	0.0	0.0	0.0

^1^Nucleotide positions are based on GenBank:FJ590753.

^2^Nucleotide positions are based on GenBank:FJ590751.

^3^Nucleotide positions are based on GenBank:FJ590752.

Baseline association of the 132L variant was performed using the logistic procedure of SAS 9.2 (SAS Institute, Cary, NC). Association analysis of other markers while accounting for 132L was performed on markers with greater than 5% minor allele frequency using the logistic procedure of SAS 9.2 with a model including the presence/absence of 132L in addition to presence/absence of the variant of interest. Additionally, similar logistic models were used to test full genotypes while accounting for 132L by performing exact conditional tests (to account for low minor allele frequencies) for genotypes of 132L and the variant of interest. Fisher's exact was used to perform specific comparison of CWD frequency among L132-bearing haplotypes.

## Results and Discussion

While it is clearly possible to have strong genetic resistance to TSEs, Rocky Mountain elk have only been shown to have the 132L substitution which confers extended incubation time with CWD but not complete resistance [9,10]. Additional variants in regulatory sites outside the open reading frame could provide increased protection, and this study used a haplotype-tagging approach to identify such variants. Specifically, this study expanded the available genomic DNA sequence and variants in the *PRNP *gene region, assessed haplotype structure in this gene region in Rocky Mountain elk, and tested common haplotype variants for association with CWD.

**Figure 1:**
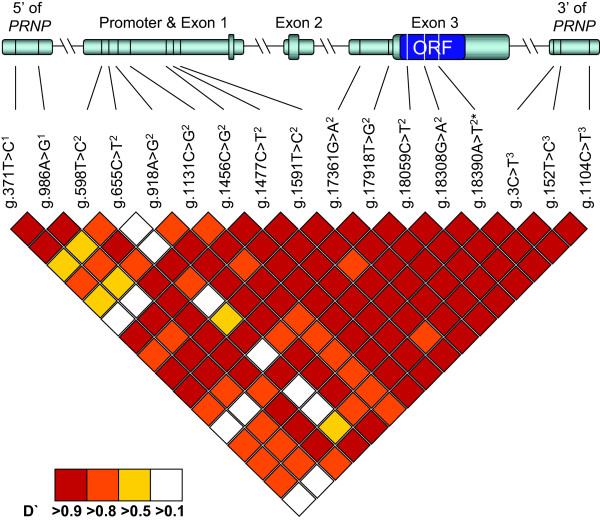
***PRNP *gene regions sequenced, haplotype-tagging markers used, and linkage disequilibrium between haplotype-tagging markers**. Sequenced regions totalling >7 kb from a genomic region spanning approximately 63 kb are shown in the upper portion of the figure as gray bars, with exons shown in larger bars. The open reading frame of *PRNP *is shown as the blue segment labelled "ORF." Haplotype-tagging markers are marked on sequenced regions as black or white bars to show position. Linkage disequilibrium between haplotype-tagging was measured by D' and is shown in the lower portion of the figure.

The current data study greatly expanded both the genomic sequence and known variants in the *PRNP *gene region of Rocky Mountain elk. As depicted in Figure 1, sequence data were generated on the proximal promoter region, all exons and splice sites, the 3'UTR, and flanking regions of the *PRNP *gene of Rocky Mountain elk. The total sequence was over 7 kb from a region spanning approximately 63 kb, of which over 40% was not previously reported to the best of our knowledge (Table 5). Further, the markers provided here approximately doubled the number of publically available variants in the *PRNP *gene region to 75 (Table 5). Most of these additional variants were obtained from the 20 elk chosen for geographic diversity. There was only one additional variant (dbSNP:ss119994812) observed from the deeper resequencing of additional case-control animals, and it had a low minor allele frequency (0.5%) in the case-control group.

**Table 5:** Sequenced regions and variants observed

*Sequenced Region*	*Total Sequence (bp)/Not Previously Reported*	*GenBank Accession*	*Total Variants Observed/Not Previously Reported*	*dbSNP Accessions*
5' of *PRNP*	1152/1152	FJ590753	9/9	ss115456948-115456956
*PRNP *Promoter & Exon 1	2526/79	FJ590751	41/15	ss119994789-119994829
*PRNP *Exon 2	469/469	FJ590751	3/3	ss120037871-120037873
*PRNP *Exon 3 (including CDS & 3' UTR)	2339/272	FJ590751	10/2	ss119994830-119994833 ss119994835-119994840
3' of *PRNP*	1168/1168	FJ590752	12/12	ss115456958-115456969
Total *PRNP *Gene Region	7654/3140	-	75/41	-

As anticipated, these 75 total variants were found to be organized into a much smaller number of haplotypes. Out of 559 elk, only 19 haplotypes were observed 3 or more times in the sample set, and only 8 haplotypes were observed at 1.0% or greater allele frequency in the total sample, with only small variations once subdivided by captive or free-ranging state of the animals (Table 4). However, since the free-ranging animals were obtained from a smaller number of locations from only one state and since captive animals have had documented human-assisted gene flow, it is difficult to make any inferences about the genetic diversity of captive versus free-ranging animals from these data. Haplotype tagging enabled representation of all common underlying variants at r^2 ^of 0.80 or greater using only 17 SNP markers, plus 5 derived genotypes composed of short multimarker haplotypes (Table 1). This represents a 77.3% reduction in variants that needed to be genotyped, which is comparable to other reports for haplotype tagging strategies in mammals [16,17]. Further, the haplotype tagging procedure required coverage of every variant in each of two animal groups, even though the variants observed in each group varied somewhat. This approach was a conservative way to ensure that all markers received coverage, but at the expense of possibly overestimating the number of markers required. The small number of haplotypes reflects relatively limited diversity in the *PRNP *gene region among Rocky Mountain elk [13], which is consistent with other reports of low genetic diversity using microsatellites in Rocky Mountain elk [22]. However, the paucity of studies based on comparable marker types precludes conclusions regarding selective sweeps in the *PRNP *region as compared to the rest of the genome. Overall, this group of tagging markers provided representation of the genetic diversity in the *PRNP *gene region for measuring local linkage disequilibrium between gene regions and for association testing with CWD.

These markers were used to investigate whether additional markers could improve the resistance provided by the previously described 132L variant (O'Rourke et al 1999). The 132L variant was underrepresented among CWD cases (P = 0.0031), occurring in cases less than half as often as the predominant genotype 132 MM (OR = 0.43, 95%CI: 0.25-0.75). However, after accounting for 132L no other variants showed significant association with CWD even on a nominal basis (P > 0.05), before any correction for multiple testing. The statistical tests specifically included comparison of CWD frequency among carriers of two haplotypes (2 and 7) that harbor L132, but no significant differences were observed (P = 0.99). Furthermore, all genotypes with any appreciable frequency showed CWD positive animals, suggesting that there is no complete resistance to CWD on the basis of common *PRNP *genotypes in these elk. While we are not aware of any epidemiological evidence to suggest that truly CWD resistant elk exist, future research could examine the possibility that complete genetic resistance to CWD does exist in Rocky Mountain elk, either because of a very low frequency *PRNP *allele or because of the influence of other genes.

## Competing interests

Katherine I. O'Rourke is the inventor of patented monoclonal antibody 99.

## Authors' contributions

SNW conceived the study, carried out the statistical analyses, and drafted the manuscript. TRS participated in sample collection and performed diagnostic pathology. JOR participated in study design, genotyping, and analysis. KIO participated in study design, sample collection, genotyping, and analysis. All authors read and approved the final manuscript.

## Supplementary Material

Additional file 1 **Amplification primers and conditions**. Primer sequences, strand directions, base positions relative to reference sequences, annealing temperatures, and included region descriptions. Click here for file

Additional file 2 **Sequencing primers**. Primer sequences, strand directions, base positions relative to reference sequences, and descriptions of regions sequenced using each primer. Click here for file
